# Pseudo-gonio synechia: An artifact on two-mirror gonioscopy

**DOI:** 10.4103/0301-4738.58473

**Published:** 2010

**Authors:** Chandra Sekhar Garudadri

**Affiliations:** LV Prasad Eye Institute, Kallam Anji Reddy Campus, LV Prasad Marg, Banjara Hills, Hyderabad – 500 034, India

**Keywords:** Goldmann gonio lens, indentation gonioscopy, pseudo-synechia, Sussman gonio lens

## Abstract

Gonioscopy is an important component of evaluation of any glaucoma patient. Goldmann two-mirror and Sussman or Zeiss four-mirror are the commonly used gonioscopes. Presence of synechia in the angle is diagnostic of angle closure disease in an occludable angle. A patient with pseudo-goniosynechia that disappeared on indentation gonioscopy with Sussman lens but persisted with manipulation gonioscopy with a Goldmann lens is reported.

Gonioscopy is an essential part of the work up for proper diagnosis and classification of glaucoma. The visualization of the angle would vary depending on the optics and the mechanics of lens used. Indentation gonioscopy has been advocated to differentiate between appositional and synechial closure of the angle.[1] Variations in gonioscopic techniques are evident from the published epidemiological studies of glaucoma.[2–7] As per the World Glaucoma Association's (WGA) consensus statement “The best lens to use remains controversial. Many specialists say that the use of a four-mirror lens is mandatory. Many others disagree. Many closed angles can be “manipulated” open using a Goldmann lens. However, a small proportion of appositionally closed angles cannot. In these cases, the use of a four-mirror lens is mandatory. For this reason, the minimum standard is a four-mirror lens.”[8] In this report we present an additional benefit of indentation gonioscopy.

## Case Report

**Figure 1:**
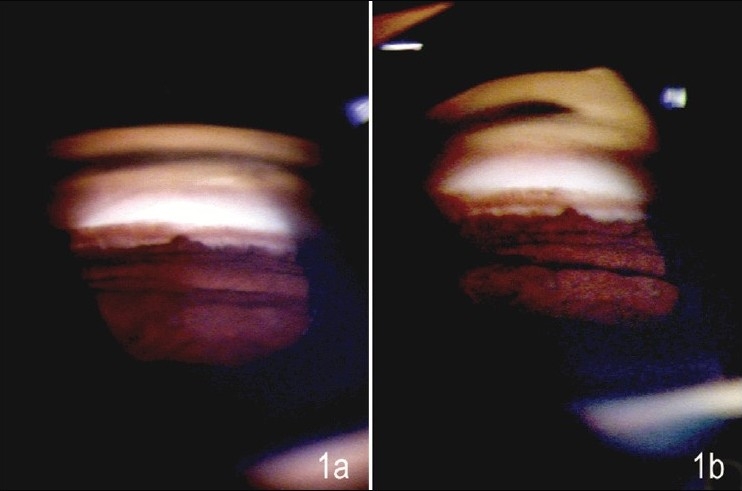
Gonioscopic appearance of the angle showing what looks like a goniosynechia in a wide-open angle a) with Goldmann lens and b) with Sussman lens

**Figure 2:**
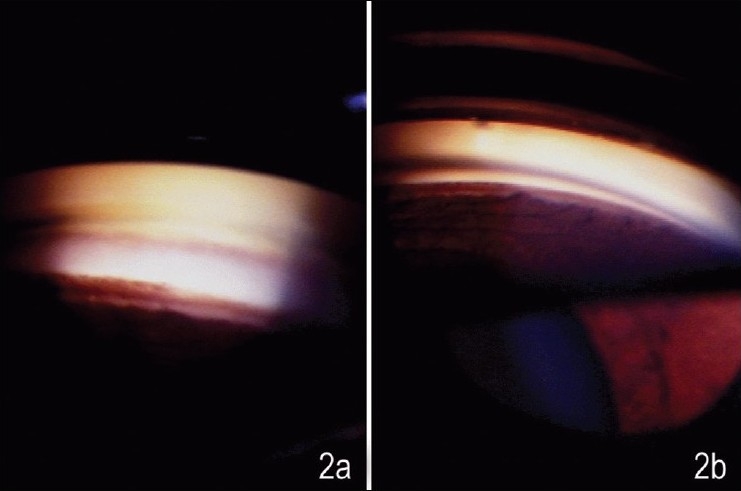
Gonioscopic appearance of the angle shown in Figure 1 on indentaion, showing the a) disappearance of the synechia with Sussman lens and b) persistence of the synechia despite manipulative gonioscopy with Goldmann lens

A 45-year-old female patient presented for a routine ophthalmic evaluation. Her visual acuity was 20/20 in each eye without correction, the intraocular pressure was 15 mm Hg in both eyes. The anterior segment examination was unremarkable, with a deep anterior chamber. She underwent gonioscopy as a part of routine complete evaluation. Fig. 1 A, B show the gonioscopic appearance of the angle with what looks like a typical goniosynechia in a wide open angle both with Goldmann (two-mirror) and Sussman (four-mirror) gonioscope. Indentation with the Sussman lens results in the disappearance of the synechia [Fig. 2A] as opposed to the Goldmann lens where in spite of the patient looking towards the mirror, with increased illumination and height of slit beam as well as “manipulation”, the “synechia” persists [Fig. 2B]. This finding we believe is due to a bulge in the periphery of the iris close to the iris insertion, which viewed end-on in gonioscopy looks like a synechia. Indentation flattens the peripheral iris and the bulge making the “synechia” disappear.

## Discussion

The WGA consensus statement reiterates the difference of opinion among specialists about the ideal gonioscope.[8] The published epidemiological studies on angle closure glaucoma have used varying gonioscopic techniques. There have been two population-based studies in Singapore and Mongolia.[2,3] In a report looking at the relationship between peripheral anterior synechia (PAS) and angle width the authors found the prevalence of synechiae in wide open angles to vary from 1.88% (Mongolia) to 3.68% (Singapore).[4] In the three epidemiological studies from South India, the prevalence of primary angle closure (PAC) varied from 0.71 to 4.32%.[5–7] It is possible that the differences in the prevalence of angle closure[5–7] may in part be due to the differences in methodology of gonioscopy. While manipulative gonioscopy can open the angle in 90-95% of cases, indentation is necessary in the rest.[1] In this case documenting the differentiation of true from pseudo-synechia was possible by indentation gonioscopy with a four-mirror and not by manipulative gonioscopy by a Goldmann gonioscope. We believe that this is an additional advantage of indentation gonioscopy, and indentation gonioscopy is essential to differentiate between pseudo and true goniosynechia.
